# Depression, sexual function and sexual quality of life in women with polycystic ovary syndrome (PCOS) and healthy subjects

**DOI:** 10.1186/s13048-023-01171-9

**Published:** 2023-05-30

**Authors:** Batool Shahraki Mojahed, Mahsa Ghajarzadeh, Razie Khammar, Zahra Shahraki

**Affiliations:** 1 Zabol university of Medial sciences, Zabol, Iran; 2 Universal Council of Epidemiology (UCE), Universal Scientific Education and Research Network (USERN), Tehran University of Medical Sciences, Tehran, Iran

**Keywords:** Polycystic ovarian syndrome, Sexual function, Iran

## Abstract

**Background:**

Women with polycystic ovary syndrome (PCOS) suffer from a wide range of psychological problems. The goal of this study is to assess depression, sexual dysfunction and sexual quality of life in women with PCO compared with healthy subjects.

**Methods:**

One hundred and six PCO cases and 106 healthy subjects enrolled. They were asked to fill out valid and reliable Persian versions of BDI (Beck depression inventory), FSFIS (Female Sexual Function Index), and sexual quality of life-Female (SQOL-F) questionnaires.

**Results:**

One hundred and six PCO cases and 106 healthy subjects enrolled. Mean BDI was significantly higher while FSFI and its subscales as well as SQOL-F were significantly lower in PCO group than controls. There were significant negative correlation between FSFO and BDI (r=-0.43, p < 0.001) and also positive correlation between FSFI and SQOL-F (r = 0.438, p < 0.001) in whole population of the study. In PCO group, 77 (72.6%) had FSFI less than 26.55 and 29 (27.4%) had score more than 26.55. mean BDI was significantly higher in the group with FSFI < = 26.55 than the other group (33 ± 14.4 vs. 12 ± 11.6, p < 0.001) while SLQL-F was significantly lower in the first group (57 ± 16 vs. 74.9 ± 17.6, p < 0.001). Linear regression analysis by considering SQOL-F as dependent and FSFI, age and BDI as independent variables showed that BDI and FSFI are independent predictors.

**Conclusion:**

Women with PCO, suffer more from depression and sexual dysfunction than healthy subjects.

## Introduction

Polycystic ovary syndrome (PCOS) is a an endocrine disease affecting 5–10% of women in reproductive age [1, 2]. Patients suffer from physical, and psychological difficulties such as hirsutism, acne, menstrual irregularity, hair loss, insulin resistance, metabolic syndrome, cardiovascular disease, and obesity as well as bipolar disorder, sexual dysfunction, depression, anxiety disorders and impaired quality of life [3–5]. Genetic, neuroendocrine, and environmental factors are considered as risk factors while the exact cause is unclear [6, 7]. PCO is one the main causes of infertility which is the cause of depression in affected cases [8, 9]. On the other hand, psychological consequences of PCO disease, and androgen excess cause sexual dissatisfaction and dysfunction [10].

Literature demonstrated that women with PCO suffer from different aspects of sexual dysfunction which affects their marital life. For instance, Dashti et al. found that desire score in women with PCO was significantly lower than healthy subjects, and was highly correlated with hirsutism [11]. Considering increasing rate of PCO all over the world, and infertility issues that affected couples face, psychologic evaluation like sexual dysfunction is recommended.

There is no report regarding sexual quality of life in women with PCO, so we designed this study to assess relationships between depression, sexual dysfunction and sexual quality of life in women with PCO compared with healthy subjects.

## Methods

This cross-sectional study was conducted in Amiralmomenin Hospital of Zabol University of Medical Sciences between March 2020 and March 2021.

### Inclusion criteria for patients were

Married women, definite diagnosis of PCO, having sexual intercourse in the last month.

### Exclusion criteria for patients were

Administration of anti-depressant medications, systematic cardiac, kidney or liver diseases, genitourinary diseases, and endocrine disorders.

### Inclusion criteria for healthy subjects were

Healthy subjects were selected from family members of enrolled patients.

Married women, no psychological disorder, no administration of anti-depressant medications, having sexual intercourse in the last month.

An expert gynecologist evaluated potential patients to find if they meet the inclusion criteria.

All participants were asked to fill informed consent forms before study entrance.

They also were asked to fill valid and reliable Persian version of BDI (Beck depression inventory), FSFIS (Female Sexual Function Index) and sexual quality of life-Female (SQOL-F) questionnaires.

BDI includes 21 questions each could be scored between 0 and 3. Total score ranges between 0 and 63.

Scores between 0 and 9 indicates no depression, scores between 10 and 18 indicates mild depression, 19–29 moderate and > 29 show severe depression [12].

FSFI has 19 questions, including six domains (desire, arousal, lubrication, orgasm, satisfaction and pain). Scores less than 26.55 indicates sexual dysfunction [13].

SQOL-F evaluates impact of sexual dysfunction including 18 questions, each containing a six-point likert scale answers (completely agree to completely disagree). The total score is sum of total scores of questions. Higher the score, higher the quality of life [14].

Data analysis was conducted using SPSS version 24 (SPSS Inc., Chicago, IL, USA). Continuous data are shown as mean ± SD and categorical data as frequencies.

Independent sample t test applied for continuous, as well as the Pearson X2 test with Fisher’s exact test used for assessment of categorical variables. Correlation coefficient was applied. Linear regression analysis conducted.

Internal consistency of the SQOL-F questionnaire was evaluated using Cronbach’s alpha.

P-value < 0.05 was considered statistically significant.

## Results

One hundred and six PCO cases and 106 healthy subjects enrolled.

Basic characteristics are summarized in Table 1.

**Table 1:** Basic characteristics of two groups

	PCO group	Control group	P value
Age	26.9 ± 5.2	27.8 ± 6.8	0.2
Partner age	38.3 ± 10	38.1 ± 8.7	0.09
Duration of marriage	8 ± 7.5	10.9 ± 8.6	0.07

The Cronbach’s alpha of SQOL-F questionnaire was 0.93.

Mean BDI was significantly higher while FSFI and its subscales as well as SQOL-F were significantly lower in PCO group than controls (Table 2).

**Table 2:** Comparison of the scores of the questionnaires

	PCO group	Control group	P value
BDI	27.3 ± 16.6	12.8 ± 8.9	< 0.001
Desire	4.1 ± 0.8	3.7 ± 1.1	0.02
Arousal	3.8 ± 1.6	3.9 ± 1.4	0.5
Lubrication	3.5 ± 1.7	4.4 ± 1.5	< 0.001
Orgasm	3.8 ± 1.8	4.5 ± 1.5	0.002
Satisfaction	3.9 ± 1.7	4.7 ± 1.2	< 0.001
Pain	2.9 ± 1.7	4.3 ± 1.4	< 0.001
Total FSFI	22.1 ± 7.8	25.7 ± 7.3	0.001
SQOL-F	62 ± 18.2	85 ± 18.8	< 0.001

There were significant negative correlation between FSFO and BDI (r=-0.43, p < 0.001) and also positive correlation between FSFI and SQOL-F (r = 0.438, p < 0.001) in whole population of the study (Table 3).

**Table 3:** The correlation coefficient between FSFO, and BDI, FSFI

	Correlation coefficient	P value
BDI	-0.43	< 0.001
FSFI	0.43	< 0.001

In PCO group, 77 (72.6%) had FSFI less than 26.55 and 29 (27.4%) had score more than 26.55. mean BDI was significantly higher in the group with FSFI < = 26.55 than the other group (33 ± 14.4 vs. 12 ± 11.6, p < 0.001) while SLQL-F was significantly lower in the first group (57 ± 16 vs. 74.9 ± 17.6, p < 0.001).

Linear regression analysis by considering SQOL-F as dependent and FSFI, age and BDI as independent variables showed that BDI and FSFI are independent predictors of SQOL-F (Table 4).

**Table 4:** Linear regression analysis considering SQOL-F as dependent and FSFI, age and BDI as independent variables

	B	P value
Age	0.184	0.26
BDI	-0.768	< 0.001
FSFI	0.574	0.001

## Discussion

To our knowledge, this is the first study, evaluating sexual quality of life in Iranian PCO women.

The results show that 72% of patients had sexual dysfunction and BDI score was significantly higher in the group with sexual dysfunction. The results also show that SQOL-F score was significantly lower in PCO group with sexual dysfunction and there was significant positive correlation between FSFI and SQOL-F which indicates that the lower FSFI score, lower SQOL-F score. We also found that FSFI score and its subscales are significantly different between PCO and control groups.

It should be noted that most women with PCO suffer from infertility due to anovulation and oligoovulation which affects psychological well-being of these cases [8] and it is clear, infertility is related with higher levels of depression and anxiety [10].

Diamond et al. reported that sexual function is not different in PCO women in comparison with healthy subjects [15] which confirmed Shafti et al. findings [16].

By including 60 women with PCO, Shakil et al. found that sexual dysfunction had significant positive correlation with depressive symptoms and negative correlation with life satisfaction level [17].

In a study which was conducted in Turkey, Deniz and Kehribar reported significant higher BDI score and lower FSFI score in PCO women than controls. They also found that PCO group with infertility problem had higher BDI and lower FSFI scores [10].

In a study by Shahraki et al., they investigated significant positive correlation between SQOL-F and total FSFI score as well as significant negative correlation between SQOL-F and BDI in Iranian infertile women. In their study, BDI and FSFI were significant predictors of SQOL-F [18].

Infertility causes anxiety and stress which affects marital status and sexual intercourse frequency [10].

In a systematic review and meta-analysis which was conducted by Loh et al., it was shown that women with PCO had 30% higher risk of having SD [19].in another systematic review and meta-analysis, Pastoor et al. found lower scores in the arousal, lubrication, orgams, and satisfaction in PCO women than controls [20].

One explanation for sexual dysfunction in these women is menstrual irregularities and subfertility cause low self-esteem and emotional distress that affects sexual quality of life and well-being [21–23].

Female sexual function is multi-dimensional and endocrine disorders, psychological distress as the result of hyper-androgenic, body dissatisfaction, obesity and dermopathy result in low self-esteem and sexual dysfunction [24].

Trent et al. found that women with PCO have 2.8 fold less sexual activity than controls [25].

It is mentioned that considering and treating psychological problems in women with PCO should be done to improve self-esteem and sexual activity.

This study had some limitations. First, it was a single center study. Second, we did not assess the hormonal status. Larger, multi-centric studies with evaluating laboratory findings are recommended.

## Conclusion

Women with PCO, suffer more from depression and sexual dysfunction than healthy subjects.
